# Knowledge of and Attitudes Toward Erosive Tooth Wear Among Students of a Portuguese University: Pilot Survey

**DOI:** 10.3390/dj13030120

**Published:** 2025-03-10

**Authors:** Patrícia Manarte-Monteiro, Maria Vittoria Buscemi, Joana Domingues, Liliana Teixeira, Bernardo Lemos, Lígia Pereira da Silva

**Affiliations:** 1 FP-I3ID, Faculty of Health Sciences, University Fernando Pessoa, 4200-150 Porto, Portugal; patmon@ufp.edu.pt (P.M.-M.); 39633@ufp.edu.pt (M.V.B.); joanad@ufp.edu.pt (J.D.); lilianat@ufp.edu.pt (L.T.); blemos@ufp.edu.pt (B.L.); 2 RISE-Health—University Fernando Pessoa, 4200-150 Porto, Portugal

**Keywords:** tooth erosion, erosive tooth wear, attitude, knowledge, university students, cross-sectional study

## Abstract

**Background/Objectives:** To assess and compare the knowledge of and attitudes toward erosive tooth wear (ETW) among university students at the Portuguese University Fernando Pessoa (UFP). **Methods:** Cross-sectional, prospective pilot study approved by the UFP-Ethics Committee for a bilingual (English and Portuguese) online self-administered questionnaire displayed to all UFP students of the 2023–2024 academic year. Two groups were recruited, dental students (DSs) and non-medical students (NMSs), based on a convenience sample of 344 students. Participants voluntarily answered demographic questions, 15 true/false/do not know questions between knowledge of ETW, and 10 positive statements to score the attitude toward ETW based on a 5-point Likert scale. Differences in scores for DSs and NMSs groups by non-parametric tests and the correlation between knowledge of and attitudes by the Pearson coefficient were considered significant for *p* < 0.05. **Results:** A total of 251 (72.9%) students participated, but only 245 (71.2%) fulfilled the survey. The knowledge and attitude scores of the DSs (Md = 12.0; IQR 11.0–13.0 and Md = 43.0; IQR 40.0–48.0) were higher (*p* < 0.001; *p* = 0.019) than those of NMSs (Md = 8.0; IQR 6.0–10.0 and Md = 41.0; IQR 38.0–46.0). The attitudes score showed similar (*p* > 0.05) results for gender, age, nationality, and curricular year. Moderate level of knowledge was higher (*p* < 0.001) for DSs (Md = 12.0, IQR 11.0–12.0) than for NMSs (Md = 10.0, IQR 9.0–10.0) students. DSs and NMSs revealed differences in attitude level distribution (*p* < 0.001). **Conclusions:** This survey highlighted the need for knowledge on ETW among NMSs, but especially the high need for measures to promote more positive attitudes toward ETW among all university students, DSs, and NMSs. It provided valuable insights into the demographic characteristics, response rate, knowledge, and attitude scores of ETW with the implementation of multicenter designs applied to similar populations being important for future research.

## 1. Introduction

Tooth erosion is a physiological process that occurs naturally over time as teeth are exposed to the oral cavity environment. However, this process can be accelerated and become disproportionate to the patient’s age, giving rise to chronic degradation of dental hard tissues due to the chemical impact of external or internal acids, excluding bacterial factors. Thus, tooth erosion becomes a pathological condition that is influenced by multiple factors and demands a comprehensive assessment for an accurate diagnosis. Successful management relies on a patient’s education for better treatment outcomes [1,2,3].

The chemical breakdown of mineralized tooth tissues by exposure to acids can be synergetic, intensified by loads such as those promoted by the tongue, cheeks, or tooth brushing, and may contribute to the accelerated loss of the demineralized tooth surfaces. Teeth are rather exposed to a combination of several types of wear, such as attrition (tooth–tooth contact wear), abrasion (teeth-to-foreign-substance contact wear), and abfraction (fatigue of the cervical part of the tooth). In addition to erosion, it is difficult to isolate these processes in the oral cavity. The overall depletion of tooth hard tissues is well-known as erosive tooth wear (ETW); however, dental erosive wear is also used for the same phenomenon [4].

Clinical characteristics include an initial reduction in tooth shining, succeeded by the flattening of convex structures. As acid exposure persists, concavities emerge on smooth surfaces, or through the presence of grooving and cupping on incisal/occlusal surfaces, tooth erosion becomes evident [5]. If neglected, this condition can eventually lead to the total depletion of tooth structure [6].

Enamel breakdown is linked to chemical factors including salivary pH, buffer capacity, titratable acidity, viscosity, and concentrations of calcium, phosphate, and fluoride in beverages and foods. These factors determine a substance’s saturation level, indicating its potential to demineralize tooth hard tissues. Substances with low pH, high titratable acidity, and strong buffer capacity pose greater erosion risk, while those with high calcium and phosphate concentrations cause less demineralization. Dentists should evaluate the erosive potential of foods and drinks, consider consumption frequency, and devise tailored preventive and dietary strategies for each patient [7].

An extrinsic factor associated with tooth erosion, the intake of acidic beverages, has been extensively examined, predominantly through in vitro and short-term in situ studies. External factors primarily involve the intake of acidic foods and beverages, such as soft drinks and fruit juices, while intrinsic factors are mainly constituted by eating disorders and gastroesophageal reflux [8]. In recent times, there has been an increase in both the overall quantity and frequency of consuming acid-containing products due to lifestyle changes [5].

In the past twenty years, researchers have increasingly focused on tooth erosion. A relatively high prevalence of erosive lesions, particularly among younger populations, has been reported, although rates often fluctuate, ranging from 20 to 45% in permanent teeth and 30–50% in primary teeth [9]. It is now widely recognized that ETW is a complex oral health issue with multiple contributing factors [2].

The prevalence and incidence of dental caries and erosion remain high [10,11]. Although their pathological mechanisms differ, they share common biological factors like salivary components and flow rate, tooth formation and structure, immune response, and individual taste preferences. These factors may be under genetic control, influencing oral disease development dynamics [12]. Studies have shown that susceptibility to erosion and caries varies among individuals exposed to similar risks [13,14,15].

Knowledge of ETW depended on individuals’ education and dental information received in the past [16]. Some authors reported a low level of awareness and knowledge of ETW among adults aged 25 to 45 years in Hong Kong [17]. A knowledge gradient regarding ETW has been identified, with dental care professionals having the most knowledge, followed by healthcare professionals and then laypersons [18]. However, the knowledge level of dental care professionals is not as high as expected. Reports from Brazil, the United Kingdom, and Yemen show that dental care professionals exhibited insufficient knowledge of ETW, which highlights the urgent need to improve education and learning outcomes on this issue worldwide [2,18,19]. Relatively little is known about the public attitudes toward ETW. Despite the common finding of increased tooth wear, especially in younger populations, during the last decade, the number of publications dealing with, for example, dental caries, surpasses studies on erosion or ETW [20].

Therefore, this study aimed to assess the knowledge of and attitudes toward ETW, of university students at the Portuguese University Fernando Pessoa (UFP). Furthermore, comparisons between the knowledge and attitudes of dental students (DSs) and non-medical students (NMSs) were carried out.

The following null hypotheses were tested: H01: DSs and NMSs have no difference in knowledge of and attitude toward ETW; H02: male and female students have no difference in knowledge of and attitude toward ETW; H03: students from several nationalities have no difference in knowledge of and attitude toward ETW; H04: knowledge of and attitudes toward ETW are not correlated for all UFP students.

## 2. Materials and Methods

### 2.1. Type of Study, Ethics Committee, Location, and Questionnaire

This cross-sectional, prospective pilot study applied an online self-administered questionnaire. UFP Ethics Committee approval (FCS/PI-501/23-3; 12 February 2024) was previously obtained.

The questionnaire was displayed to all UFP students in both Portuguese and English [21] languages to evaluate the knowledge of and attitudes toward erosive tooth wear among different nationalities of students. All students were given the opportunity to respond to each question only once according to their language of choice, either the Portuguese translation or the English version of the questionnaire.

The questionnaire includes demographic questions concerning gender, age, scientific field, faculty, academic year, and nationality. The remainder of the questionnaire was divided into 2 sections to assess the knowledge and attitudes.

The knowledge section of the questionnaire included 15 true/false/do not know questions on knowledge of erosive tooth wear (items K1–K15; or C1–C15 in the Portuguese version). The participants were asked to answer each question with “true”, “false”, or “do not know”. Each correct response received a score of 1, while an incorrect or “do not know” answer received a score of 0. The knowledge scores ranged from 0 to 15 and were calculated by summing the scores for the items in the knowledge section. The knowledge score described the respondent’s knowledge of erosive tooth wear; higher sum scores indicated more accurate knowledge.

The attitude section of the questionnaire collected information using 10 positively framed statements (items A1–A10). The attitude score was based on a 5-point Likert scale, an instrument widely used in research on opinions, beliefs, and attitudes. For the five response options, items were assigned 1 point for “strongly disagree”, 2 points for “disagree”, 3 points for “neither agree nor disagree”, 4 points for “agree”, and 5 points for “strongly agree”. The attitude scores ranged from 10 to 50 and were calculated by summing the scores for the items in the attitude section of the questionnaire. The attitude score described the respondent’s attitude toward erosive tooth wear; higher scores indicated a more positive attitude.

### 2.2. Population and Sample

The questionnaire was distributed to all university students via e-mail through an official university mailing list, considering the two majors’ academic scientific fields: dental students (DSs) and non-medical students (NMSs).

According to the estimated population of students (N = 3281) in the academic year 2023–2024, a convenience sample should comprise 344 students, with a margin of error of 0.05. The sample size was calculated based on a formula by Cochran stating. Considering the response rates for dental erosion surveys (45–79%), a sample of 237 to 341 (95% confidence level) university students was considered sufficient.

All students of the 2023–2024 academic year were invited to participate. The questionnaire was displayed online on 12 March 2024 and 2 May 2024. DSs and NMSs were recruited.

#### 2.2.1. Inclusion Criteria

All students at University Fernando Pessoa (UFP) who freely agreed to take part in this survey did so by fully completing the online self-administered questionnaire and after signing the informed consent.

#### 2.2.2. Exclusion Criteria

UFP students who did not agree to participate in the study or who did not complete the entire survey. External students of UFP, from other health institutions or high schools.

### 2.3. Complications and Risks

No risks for participants. An introduction text explaining the study design, the consent form, and the questionnaire was displayed by email to the UFP students using the online Google Form survey system by the UFP Communication and Image Office, the research principal investigator (P.M.-M.), and a UFP student (M.V.B.).

The participation of the students was entirely voluntary, and the data were anonymously collected and analyzed, as the participants were not requested to provide their names or any other information that could be used to personally identify them.

### 2.4. Data Confidentiality and Research

Participation was completely voluntary and free of charge. The analytical results were completely confidential, and at no time will the identities of the participants be revealed or disclosed. Identity and personal data were not disclosed in accordance with current data protection legislation. The description of the terms of confidentiality of data and research was explained in the informed consent applied to the questionnaire for this research.

### 2.5. Reliable and Scientifically Recognized Alternative Acts/Interventions and Risk of Non-Monitoring

This pilot study used a questionnaire in both Portuguese and English languages. The English version was provided by Professor Dent-Wei Hong to the research principal investigator [21].

The questionnaire was translated and adapted to the Portuguese language, and the face validity was confirmed by 3 academic-senior experts (P.M.-M., L.T., and J.D.) in the field of preventive and conservative dentistry. Essential revisions of the included items were made based on feedback from the consultation by the authors [21].

### 2.6. Statistical Analysis

The levels of knowledge and attitudes were defined according to Bloom’s initial thresholds: scores above 80% of the total score indicated a high level of knowledge or a positive attitude; scores between 60 and 80% of the total scores indicated a moderate level of knowledge or a neutral attitude; and scores below 60% of the total score indicated a low level of knowledge or a negative attitude. Therefore, knowledge scores <9, between 9 and 12, and >12 indicated low, moderate, and high levels of knowledge on ETW, respectively. Attitude scores <34, between 34 and 42, and >42 indicated negative, neutral, and positive attitudes toward ETW, respectively.

Demographic data (gender, age, nationality, curricular year, and DSs versus NMSs) were subjected to descriptive statistical analysis according to students’ age, knowledge, and attitude scores. To assess the age variable, a cut-off value was assumed according to the sample’s median (Md) age and interquartile ratio (IQR) values. Data distribution was graphically assessed by histograms and by Shapiro–Wilk hypothesis test analysis.

The Mann–Whitney U test was applied to compare knowledge and attitude scores by gender, age, nationality, curricular year, and DSs/NMSs groups. Knowledge (weak, moderate, and high) and attitudes (negative, neutral, and positive) levels for the DSs and NMSs groups were compared by Kruskal–Wallis and Mann–Whitney U tests. Association analysis of knowledge correct answers (scored as 1) and neutral/positive attitudes (scored as Likert 3, 4, or 5) for DSs or NMSs groups was tested through the Pearson Chi-square test.

The correlation between knowledge and attitude scores was assessed using Pearson’s correlation coefficient.

All data were computerized and analyzed using IBM SPSS Statistics for Macintosh, Version 29.0.2.0. Statistically significant differences were considered for *p* values < 0.05.

## 3. Results

A total of 245 UFP students answered the survey after signing the informed consent. Six more students returned the questionnaire and declined to participate. The survey response and participation rates were 71.2% and 72.9%, respectively, considering the convenience sample of all UFP students (N = 344). A normal distribution was not found (*p* < 0.05) for all the variables tested. Among the 245 students, 139 (56.7%) were DSs and 106 (43.3%) were NMSs. Age varied from 18 to 75 years old (Md = 22; IQR 20–25). Of those, 164 (66.9%) were females, 225 (91.8%) had European Union (EU) nationalities, and 139 (56.7%) belonged to the 1st, 2nd, and 3rd curricular years of the UFP training course. Table 1 represents the age and gender, nationality, curricular year, and DSs/NMS distribution of the participants.

The knowledge score and attitude score of the DSs (Md = 12.0; IQR 11.0–13.0 and Md = 43.0; IQR 40.0–48.0) were higher (*p* < 0.001 and *p* = 0.019) than those of NMSs (Md = 8.0; IQR 6.0–10.0 and Md = 41.0; IQR 38.0–46.0), respectively. Furthermore, the knowledge of male students (Md = 12.0, IQR 9.0–13.0; *p* = 0.001), students older than 22 years (Md = 12.0, IQR 10.0–13.0), and students with curricular year > 3 years (Md = 12.0; IQR 11.0–13.0; *p* < 0.001) showed significantly higher scores for ETW than female students, students younger than 22 years old, and students with curricular years ≤ 3 years, respectively. Similar ETW knowledge scores were found for students of different nationalities (*p* = 0.330). The attitude score of the DSs was higher than those of NMSs (*p* = 0.019). However, no differences (*p* > 0.05) were found for ETW attitude scores for gender, age, nationality, and curricular year (Table 2).

The DSs showed higher scores for 12 (*p* < 0.05) of the 15 knowledge questions. DSs and NMSs tendencies showed similar (*p* > 0.05) knowledge of ETW for the K3, K4, and K14 questions in the knowledge section of the survey (Table 2). The correct answer to the K3 question was more prevalent (*p* < 0.001) in the DSs (85.5%) group than in the NMSs group.

In the DSs group, high (Md = 13.0, IQR 13.0–14.0) and moderate (Md = 12.0, IQR = 11.0–12.0) levels of knowledge were similar (*p* > 0.05) for 37.4% (n = 52) and 54.7% (n = 76), respectively, but significantly different (*p* = 0.045) of 7.9% (n = 11) with a weak (Md = 6.0, IQR 0–8.0) level of knowledge (Table 3). The NMSs group revealed similar (*p* > 0.05) high (Md = 13.5, IQR 13.0–NA) and moderate (Md = 10.0, IQR 9.0–10.0) levels of knowledge only for 1.9% (n = 2) and 42.5% (n = 45), respectively, but significantly different (*p* = 0.045) of 55.7% (n = 59) with weak (Md = 6.0, IQR 4.0–7.0) level of knowledge. Moderate level of knowledge was significantly higher (*p* < 0.001) for DSs (Md = 12.0, IQR 11.0–12.0) than for NMSs (Md = 10.0, IQR 9.0–10.0) students. No differences were found between DSs and NMSs participants on behalf of the weak and the high levels of knowledge (both, *p* > 0.05) of knowledge (Table 3).

The DSs showed higher median values, and the frequency of the Likert scale scored 3 to 5 (*p* < 0.05) in all attitude scores except for A1 and A2 questions of the survey attitude section (Table 4).

DSs and NMSs revealed differences (*p* < 0.001) for attitude level distribution. In the DSs group, 56.1% (n = 78) of participants showed significant (*p* < 0.04) positive (Md = 48.0, IQR 45.0–50.0) attitudes for ETW when compared to 40.3% (n = 56; *p* < 0.04) and 3.4% (n = 5; *p* < 0.001) of neutral (Md = 40.0, IQR 38.0–40.0) and negative (Md = 29, IQR 29.0–31.0) attitudes, respectively. NMSs group, 53.8% (n = 57) registered neutral (Md = 39.0, IQR 36.0–41.0) attitude level for ETW when compared with 40.6% (n = 43; *p* = 0.04) and 5.7% (n = 6; *p* < 0.001) with positive (Md = 49.0, IQR 44.0–50.0) and negative (Md = 32.0, IQR 30.0–33.0) attitudes, respectively. No differences (*p* > 0.05) were found for DSs and NMSs when comparing each attitude (negative, neutral, and positive) level (Table 5).

The attitude score only suggested a positive correlation (Pearson correlation coefficient; r = 0.101; *p* > 0.05) with the knowledge score. Furthermore, the DSs group also suggested (r = 0.031; *p* > 0.05) a positive correlation between attitude and knowledge scores. For the NMSs group, a tendency (*p* > 0.05) to a negative (r = −0.001) correlation between attitude and knowledge scores.

## 4. Discussion

Based on the pilot study outcomes, the null hypothesis that dental students (DSs) and non-medical students (NMSs) of UFP have no difference in knowledge of and attitude toward ETW was rejected. The knowledge and attitude scores of the DSs were significantly higher than those of NMSs. There was a notable disparity in ETW’s moderate level of knowledge between DS and NMS students. Also, the moderate and high levels of knowledge in DSs were higher and distinct from those with weak levels of knowledge. However, for NMSs, the weak level of knowledge of ETW was more prevalent than the moderate and high levels. Moreover, DSs showed more frequent positive attitudes than those with neutral and those with negative attitudes. Similarly, results were detected for NMSs. However, no differences were detected between DSs and NMs for each attitude level toward ETW. Al-Ashtal et al. [2] and Hong et al. [21] assessed knowledge and attitudes toward dental erosion among dental professionals and students. Those authors found that only half of the respondents had in-depth knowledge of ETW. Hong et al. [21] pointed out that dental students had better knowledge and more positive attitudes toward erosion than students of other disciplines. The specific field of education appeared to be a determining factor in the perception and knowledge of ETW. Hong et al. [21] pointed out that participants with dental training demonstrated greater knowledge, suggesting that interprofessional education could extend this awareness beyond the healthcare sector. Verploegen and Schuller [16] and Søvik et al. [22] underlined the importance of educating young people on the effects of acidic foods and drinks and dental erosion, while Al-Ashtal et al. [2] emphasize the need for dental professionals to be more aware of these factors. On the other hand, Verploegen & Schuller [16] and Dynesen et al. [23] investigated knowledge levels and the need for specific information on ETW. This study found that the majority of young adults preferred to receive information from oral health professionals. Dentists should acquire empathetic communication skills toward patients with eating disorders [23].

Our null hypothesis that tested that female and male students have no difference in knowledge of and attitude toward ETW was partially rejected. Students’ gender and age influenced the knowledge scores, but no differences were detected for gender regarding the attitude scores. Male students, those who are more than 22 years old and with more than 3 curricular years in UFP, showed significantly higher knowledge scores than females, students aged 22 years or younger, and those with less than 3 curricular years in UFP. However, attitudes toward ETW scores were similar by gender, by student age, and by the university curricular year frequency. Furthermore, the null hypothesis that students’ nationality, which involved EU and non-EU students’ nationalities at UFP, has no difference in levels of knowledge and attitudes toward ETW was accepted since no significant differences within those student groups were observed.

Our findings revealed that the attitude and knowledge scores were not significantly correlated, though a slight positive tendency was observed for all UFP students and for DSs, and a slight negative correlation tendency for the NMSs group.

This pilot study among UFP university students provided valuable insights into demographic characteristics, response rate, and comparison of knowledge and attitude scores regarding ETW among DSs and NMSs. Those findings highlighted the need for knowledge of ETW among NMSs, but especially the high need for measures to promote more positive attitudes toward ETW among all this community, including some investment in positive attitudes of DSs toward all university students’ communities.

The study conducted by Schmidt and Huang [24] emphasized the importance of oral health education and the changing of acid-drinking behaviors, suggesting that health professionals should actively educate people on ETW. The importance of educating young adults, as those with higher levels of education and those who received dental professionals’ information, showed a better understanding of the subject [16]. In addition, a significant correlation was identified between the severity of ETW and education level [25], which highlights the importance of focusing information efforts on less-educated young adults. However, it is important to note that other studies [26] found no significant correlation between parental education and ETW in children.

The outcomes of twelve cross-sectional studies [2,16,21,22,23,24,25,27,28,29,30,31] qualitatively indicate the need for additional research on etiological factors that may be associated with ETW and for promoting knowledge of, attitude, and awareness toward ETW in several populations.

The main outcomes of several studies, though different evaluation methodologies applied, evidenced the need for attitudes and, in some cases, the urgent need for enhancing awareness and knowledge of ETW in several communities [2,16,21,22,23,24,25,28,29,30,31]. For the pertinence of this issue, cross-sectional surveys have been applied in several communities and in young populations of different countries, such as children (12 years old) and young adults (18 to 25 years old) in Hungary, the United Kingdom, Finland, Latvia, Estonia, the Netherlands, and Germany [16,25]. In adolescents of public and private high schools of northeastern Brazil [28], in runners/amateur athletes of Rio de Janeiro, Brazil [27], 18-yr-old subjects of Oslo Public Dental Service clinics, Norway [31], in the 1st year (aged 18–21 years old) training of University campus of Hong Kong [29], in the fifth year dental students of University of Science and Technology, Sanaa, Yemen [2], in dental, medical, and non-medical university students of two major Chinese universities [21], in students of Australian University [24]. Furthermore, in a consecutive adult (18 to 46 years old) sampling of the Health Centre in San Bernardo, Chile [30].

ETW is a multifactorial disease that can be caused by intrinsic (e.g., gastric reflux and excessive vomiting) and/or extrinsic (e.g., acidic foods and drinks and acid occupational vapors) factors [21]. It is important that dental care professionals are able to diagnose the condition as early as possible, to identify the possible etiology of the erosive damage, and also to understand the specific host defense factors of importance in each case [2].

In Portugal, despite the scientific and professional community’s growing interest in research on this topic, there are few data on ETW in adults, and as far as the authors know, no student or other population insights have been collected to investigate the risk factors, the knowledge of, the awareness, or attitudes toward this oral condition. Therefore, this pilot survey emphasized the need for evidence of knowledge and attitudes of different student profiles attending several scientific fields of higher education, as the included ones from humanities and social sciences, science and technology, pharmaceutical sciences, and nutrition sciences, in regard to ETW, an oral condition with multifactorial etiology, of endogenous (e.g., gastro-esophageal reflux) or exogenous origins [32,33], associated or not with the diversity of lifestyles and behaviors or consumption practices. The growing concerns and promotion of healthy lifestyles among the younger population and university students can influence dietary habits and the consumption of products with low sugar content but high acidic composition. As recently stated by Saads Carvalho and Lussi [7], current reviews of outcomes revealed that frequent consumption of carbonated/soft drinks, vitamin C, natural fruit juices, and acidic snacks or sweets was significantly associated with more ETW.

The individual’s environment, prescription medications, dietary choices, and lifestyle, all of which can contribute to ETW [34]. A significant correlation was observed between daily consumption of erosive beverages and dental erosion [25]. Similarly, a lack of awareness of dental erosion can influence the perception of ETW/dental erosion severity, as pointed out by some authors [29]. In terms of clinical implications, dental erosion/ETW can cause pain, sensitivity, and functional and esthetic limitations. However, access to optimal ETW management remains a challenge, particularly in developing countries, due to limited costs and resources [21].

In the present pilot study, the self-administered questionnaire response and participation rates were 71.2% (n = 245) and 72.9% (n = 251), respectively, and were considered sufficient and representative of the studied population, the UFP DS and NMS students. This cross-sectional pilot study was based on a convenience sample (n = 344; 95% confidence interval and 5% margin of error), considered for 50% of response distribution, that corresponded to the largest sample size, calculated by the Sample Size Calculator software (Inc.RaoSoft^®^, Seattle, WA, USA) (http://www.raosoft.com/samplesize.html accessed on 30 December 2024) and, based on the formula by Cochran, stated. Considering the self-administered response rates for dental erosion of 45–79% in similar surveys [2,16,19,21,31], the sample for this trial could have varied between 341 and 237 individuals, respectively. A recent online questionnaire applied at an Australian university reported a 96.3% completion rate [24].

The ETW questionnaire applied in this pilot study was provided in the English language by the authors of a survey conducted among Chinese university students, at two major universities in Fujian Province, China [21]. After the authors’ permission, the questionnaire was translated into Portuguese by two dental academic teachers (L.P.D.S. and B.L.) and then reviewed by three independent bilingual senior experts, dental academic teachers (P.M.-M., L.T., and J.D.). The two versions were pretested for critical assessment and technical functionality on 25 UFP dental teachers and students (not included in the final sample of the pilot study), who fulfilled the questions individually for the detection of possible discrepancies of language or contextual inconsistencies. Some translated questions were reviewed. Different guidelines and theoretical approaches to achieve content validation of instruments are discussed in the literature [35]. Though the statistical validity and reliability were not performed for the Portuguese inquiry version, the translation and review procedures were performed to ensure that when fulfilling the questionnaire, the students had no doubts and had access to the equivalent questions, such as language and cultural aspects. As UFP students have bilingual learning (English and Portuguese), the questionnaire was provided to all students in both languages. This questionnaire design was replicated [21] for several reasons such us, standardization (a consistent design across different studies or applications allows for standardized data collection and helps to ensure that the same concepts or variables are measured the same way each time), for improving comparisons (allows to compare results across different populations, time periods, or settings, crucial for tracking trends or differences in behavior, attitudes, or perceptions over time), for legal and ethical compliance (ensures transparency in how data were collected and measured, important for results sharing, for example, with stakeholders, regulatory bodies, or the public; It helps maintain trust in the research process and its outcomes; also ensures that respondents were treated fairly and that their responses were interpreted correctly, minimizing any potential biases or misunderstandings in the data collection process) and also, for a wider applicability (replication across different contexts, regions, or populations allows researchers to assess the generalizability of their findings) helping to ensure that the results are applicable to a broad audience and are not limited to a specific sample or setting.

The main purposes of some cross-sectional studies focused on assessing the knowledge, awareness, or attitudes toward ETW [2,16,21,23,24,31], on the prevalence and severity of ETW [24,25,27,29,30,31], as well as the evaluation of both erosion and dental decay [28,29]. Also, two main designs of cross-sectional surveys for ETW data collection in younger populations and students can be literally evidenced: only questionnaire [2,16,21,23,24,27], only clinical examination [31], or both clinical observation and insights questionnaires [22,25,28,29,30].

With regard to ETW prevalence, Jász and Szőke’s [25] study revealed a higher prevalence of dental erosion in adolescents attending private schools compared to those in public schools, underscoring the influence of socio-economic status on this disparity. These findings align with European research, which has linked a lower prevalence of dental erosion to higher socio-economic status, probably due to healthier diets. However, in developing countries, lower socio-economic status was associated with lower dental erosion prevalence, probably due to limited access to expensive, erosive soft drinks.

Some limitations of this study can be reported, such as the statistical validity and reliability of the Portuguese version that was not yet performed, the non-inclusion of other higher education students or other health groups, such as those in the medical field, and even from other universities (public or other private) in different regions of Portugal, or other populations associated with teaching/training and student learning outcomes; the relatively low participation rate of non-EU students (only 8.2%, n = 20) compared to EU students (91.8%; n = 225), which may have contributed to the present outcomes. Furthermore, self-reporting bias could influence some of the results. Furthermore, self-reporting bias could have influenced some of the results. So, the findings of this survey should not be extrapolated for other contexts than the studied one. However, it underlined the need for policies to raise awareness of this public health and clinical condition, being important in the near future to gather insights from more students, if possible, on a national scale but also from other stakeholders, like their teachers as agents of transmitting knowledge and attitudes with influence in the students’ learning outcomes. In addition to the application of self-assessment surveys, it is also essential to carry out an adequate clinical assessment of hard dental structures and their respective records, by acceptable instruments and methodologies such as the Basic Erosive Wear Examination (BEWE) [36] or other indexes for detection of scientific and clinical needs as some trials had performed [22,25,28,29,30].

This work helped to highlight the worth of education and prevention strategies as well as the integration of dental professionals’ interventions in the community and in the multidisciplinary management of related diseases, such as eating or other disorders.

## 5. Conclusions

The outcomes of this pilot study highlighted need for knowledge on ETW among NMSs but especially the high need for measures to promote more positive attitudes toward ETW into all university students, DSs, and NMSs. This survey provided valuable insights among the demographic characteristics, response rate, and comparison of knowledge and attitude scores regarding dental erosion/ETW among DSs and NMSs, being important in future research on the implementation of multicenter designs applied to similar populations in Portugal, as well as the adequate clinical assessment of hard dental structures and their respective records, by acceptable instruments and methodologies such as the Basic Erosive Wear Examination.

## Figures and Tables

**Table 1:** Student’s (n = 245) demographic description by age/gender, nationality, curricular year of university training, and by groups (DSs and NMSs), for knowledge and attitude scores. The Mann–Whitney U test was applied to assess possible differences in knowledge and attitude scores concerning different groups (i.e., gender, age, nationality, curricular year, and DSs versus NMSs).

Students Characteristics (n; %)	Age Median (P_25_–P_75_)	Knowledge Score Median (P_25_–P_75_)	*p*-Value ^1^	Attitude Score Median (P_25_–P_75_)	*p*-Value ^1^
**Gender**					
Male (n = 81; 33.1)	24.0 (21.5–28.0)	12.0 (9.0–13.0)	0.001	42.0 (39.0–48.0)	0.808
Female (n = 164; 66.9)	22.0 (20.0–24.0)	10.0 (7.0–12.0)		43.0 (38.3–48.0)	
**Age** (minimum-maximum: 18–75 years old)					
≤22 years (n = 124; 50.6)	22.0 (20.0–25.0)	9.0 (6.0–11.0)	<0.001	43.0 (39.3–48.8)	0.628
>22 years (n = 121; 49.4)		12.0 (10.0–13.0)		42.0 (39.0–47.5)	
**Nationality**					
EU ^2^ (n = 225; 91.8)	22.0 (20.0–24.0)	11.0 (8.0–12.0)	0.330	43.0 (39.0–48.0)	0.161
Non-EU (n = 20; 8.2)	38.5 (32.3–54.5)	10.0 (8.0–11.0)		39.5 (36.3–49.0)	
**Curricular year**					
≤3 years (n = 134; 54.7)	20.0 (19.0–22.0)	9.0 (6.0–11.0)	<0.001	42.0 (39.0–48.0)	0.791
>3 years (n = 111; 45.3)	24.0 (23.0–27.0	12.0 (11.0–13.0)		43.0 (39.0–48.0)	
**DSs ^3^ and NMSs ^4^**					
DSs (n = 139; 56.7)	24.0 (22.0–27.0)	12.0 (11.0–13.0)	<0.001	43.0 (40.0–48.0)	0.019
NMSs (n = 106; 43.3)	20.0 (19.0–22.0)	8.0 (6.0–10.0)		41.0 (38.0–46.0)	

^1^ *p*-value by Mann–Whitney U test; ^2^ European Union; ^3^ dental students; ^4^ non-medical students.

**Table 2:** Knowledge of ETW scores comparison of dental students (DSs) and non-medical students (NMSs). Pearson Chi-square test was applied to assess possible differences in knowledge score results of DSs versus NMSs groups regarding correctly answered questions; the Mann–Whitney U test was applied to assess possible differences in knowledge global scores between the two groups.

Item Number–Content	Knowledge Score “Answered Correctly” n (%)		*p*-Value ^1^	Knowledge Global Score Median (P_25_–P_75_)		*p*-Value ^2^
	DSs ^3^	NMSs ^4^		DSs ^3^	NMSs ^4^	
K1—Erosive tooth wear is a form of cavities and tooth decay	117 (84.2)	23 (21.7)	<0.001	1.0 (1.0–1.0)	0.0 (0.0–0.0)	<0.001
K2—Erosive tooth wear is caused by bacteria	116 (83.5)	19 (17.9)	<0.001	1.0 (1.0–1.0)	0.0 (0.0–0.0)	<0.001
K3—Erosive tooth wear is an irreversible disease	56 (40.3)	56 (52.8)	0.051	0.0 (0.0–1.0)	1.0 (0.0–1.0)	0.051
K4—One leading cause of tooth wear is acid in our food and drinks	119 (85.6)	83 (78.3)	0.136	1.0 (1.0–1.0)	1.0 (1.0–1.0)	0.137
K5—Saliva is one of the most important defense mechanisms against erosion	119 (85.6)	59 (55.7)	<0.001	1.0 (1.0–1.0)	1.0 (0.0–1.0)	<0.001
K6—Erosive tooth wear can occur if you often work in acidic environments	106 (76.3)	28 (26.4)	<0.001	1.0 (1.0–1.0)	1.0 (0.0–1.0)	<0.001
K7—Erosive tooth wear can occur if you often must vomit	132 (95.0)	57 (53.8)	<0.001	1.0 (1.0–1.0)	1.0 (0.0–1.0)	<0.001
K8—Brushing your teeth immediately after consuming acidic food or drinks may make erosive tooth wear worse	114 (82.0)	42 (39.6)	<0.001	1.0 (1.0–1.0)	0.0 (0.0–1.0)	<0.001
K9—Drinking before going to bed is a risk factor for developing erosive tooth wear	91 (65.5)	32 (30.2)	<0.001	1.0 (0.0–1.0)	0.0 (0.0–1.0)	<0.001
K10—Drinking immediately after strenuous exercise increases a person’s risk for erosive tooth wear	99 (71.2)	51 (48.1)	<0.001	1.0 (0.0–1.0)	0.0 (0.0–1.0)	<0.001
K11—Erosive tooth wear may lead to pain and sensitivity	133 (95.7)	88 (83.0)	<0.001	1.0 (1.0–1.0)	1.0 (0.0–1.0)	<0.001
K12—Erosive tooth wear can lead to the progressive loss of the surface of the tooth	134 (96.4)	76 (71.7)	<0.001	1.0 (1.0–1.0)	1.0 (0.0–1.0)	<0.001
K13—Drinking a whole bottle of soda in several sittings rather than in just one sitting decreases a person’s risk for erosive tooth wear	85 (61.2)	50 (47.2)	0.029	1.0 (0.0–1.0)	0.0 (0.0–1.0)	0.030
K14—Using a fluoride toothpaste will prevent erosive tooth wear	99 (71.2)	68 (64.2)	0.239	1.0 (0.0–1.0)	1.0 (0.0–1.0)	0.240
K15—Using a straw when you drink soda may help avoid erosive tooth wear	83 (59.7)	49 (46.2)	0.036	1.0 (0.0–1.0)	0.0 (0.0–1.0)	0.036

^1^ *p*-value by Pearson Chi-square test; ^2^ *p*-value by Mann–Whitney U test; ^3^ dental students; ^4^ non-medical students.

**Table 3:** Knowledge of ETW levels distribution of dental (DSs) and non-medical (NMSs) students. Kruskal–Wallis test was applied to assess possible differences in knowledge levels within each group (DSs and NMSs); the Mann–Whitney U test was applied to assess possible differences in knowledge levels between DSs and NMSs groups.

Student Study Groups	Knowledge Levels						Total Median (P_25_–P_75_)	*p*-Value ^1^
	Weak		Moderate		High			
	n (%)	Median (P_25_–P_75_)	n (%)	Median (P_25_–P_75_)	n (%)	Median (P_25_–P_75_)		
DSs (n = 139)	11 (7.9)	6.0 (0.0–8.0) _a_	76 (54.7)	12.0 (11.0–12.0) _b_	52 (37.4)	13.0 (13.0–14.0) _b_	12.0 (11.0–13.0)	**<0.001**
NMSs (n = 106)	59 (55.7)	6.0 (4.0–7.0) _a_	45 (42.5)	10.0 (9.0–10.0) _b_	2 (1.9)	13.5 (13.0–NA ^3^) _b_	8.0 (6.0–10.0)	**<0.001**
***p*-value ^2^**		0.948		**<0.001**		0.771	**<0.001**	

^1^ *p*-value by Kruskal–Wallis test; ^2^ *p*-value by Mann–Whitney U test; ^3^ not applicable. a,b—different letters show significant differences according to the Mann–Whitney U test. Knowledge scores: <9, between 9 and 12, and >12 indicated weak, moderate, and high levels of knowledge of erosive tooth wear, respectively.

**Table 4:** Attitude toward ETW, scores comparison of dental students (DSs) and non-medical students (NMSs). The Pearson Chi-square test was applied to assess possible differences in attitude score results of DSs versus NMSs groups; the Mann–Whitney U test was applied to assess possible differences in attitude global scores between the two groups.

Item Number-Content	Attitude Scored as 3, 4 or 5 n (%)		*p*-Value ^1^	Attitude Global Score Median (P_25_–P_75_)		*p*-Value ^2^
	DSs	NMSs		DSs	NMSs	
A1—I think oral health is just as important as general health	139 (100.0)	106 (100.0)	0.264	5.0 (4.0–5.0)	5.0 (4.0–5.0)	0.274
A2—I think prevention is better than a cure	138 (99.3)	106 (100.0)	0.161	5.0 (5.0–5.0)	5.0 (4.0–5.0)	0.053
A3—It is essential to visit a dentist at least every half year for a regular dental check-up	139 (100.0)	104 (98.1)	0.086	5.0 (4.0–5.0)	5.0 (3.0–5.0)	**0.031**
A4—I would think that it is bad if I learned that my teeth had been damaged by acid	137 (98.6)	104 (98.1)	**<0.001**	5.0 (4.0–5.0)	4.0 (4.0–5.0)	**<0.001**
A5—It is worth spending more time and energy on studying knowledge about erosive tooth wear	138 (99.3)	100 (94.3)	**<0.001**	4.0 (4.0–5.0)	4.0 (3.0–4.0)	**<0.001**
A6—I am concerned with whether or not the drinks I consume are acidic	133 (95.7)	77 (72.7)	**<0.001**	4.0 (4.0–5.0)	3.0 (2.0–4.0)	**<0.001**
A7—I am concerned with whether or not a toothpaste contains fluoride	136 (97.8)	81 (76.5)	**<0.001**	4.0 (4.0–5.0)	4.0 (3.0–4.0)	**<0.001**
A8—To prevent erosive tooth wear, I would change my dietary habits (such as controlling my consumption of soft drinks)	136 (97.8)	97 (91.6)	**0.001**	4.0 (4.0–5.0)	4.0 (3.0–5.0)	**<0.001**
A9—To prevent erosive tooth wear, I would change my behavior habits (such as drinking from a straw)	139 (100.0)	98 (92.5)	**<0.001**	4.0 (4.0–5.0)	4.0 (3.0–5.0)	**<0.001**
A10—I would see a doctor immediately if I learned that my teeth had been damaged by acid	137 (98.6)	101 (95.3)	**0.009**	5.0 (4.0–5.0)	4.0 (4.0–5.0)	**0.001**

^1^ *p*-value by Pearson Chi-square test; ^2^ *p*-value by Mann–Whitney U test.

**Table 5:** Attitude toward ETW levels distribution of dental students (DSs) and non-medical students (NMSs). The Kruskal–Wallis test was applied to assess possible differences in attitude levels within each group (DSs and NMSs); the Mann–Whitney U Test was applied to assess possible differences in attitude levels between DSs and NMSs groups.

Student Study Groups	Attitude Levels						Total Median (P_25_–P_75_)	
	Negative		Neutral		Positive			*p*-Value ^1^
	n (%)	Median (P_25_–P_75_)	n (%)	Median (P_25_–P_75_)	n (%)	Median (P_25_–P_75_)		
DSs (n = 139)	5 (3.4)	29.0 (29.0–31.5) _a_	56 (40.3)	40.0 (38.0–40.0) _b_	78 (56.1)	48.0 (45.0–50.0) _c_	43.0 (40.0–48.0)	**<0.001**
NMSs (n = 106)	6 (5.7)	32.0 (30.0–33.0) _a_	57 (53.8)	39.0 (36.0–41.0) _b_	43 (40.6)	49.0 (44.0–50.0) _c_	41.0 (38.0–46-0)	**<0.001**
***p*-value ^2^**		0.126		0.387		0.890	**0.019**	

^1^ *p*-value by Kruskal–Wallis test; ^2^ *p*-value by Mann–Whitney U test. a–c—different letters show significant differences according to the Mann–Whitney U test. Attitude scores <34, between 34 and 42, and >42 indicated negative, neutral, and positive attitudes toward erosive tooth wear, respectively).

## Data Availability

The raw data supporting the conclusions of this article will be made available by the authors on request.
